# Microfluidic Thermal Flowmeters for Drug Injection Monitoring

**DOI:** 10.3390/s22093151

**Published:** 2022-04-20

**Authors:** Il Doh, Daniel Sim, Steve S. Kim

**Affiliations:** 1 Medical Metrology Team, Safety Measurement Institute, Korea Research Institute of Standards and Science (KRISS), Daejeon 34113, Korea; il.doh@kriss.re.kr; 2 Department of Applied Measurement Science, University of Science and Technology (UST), Daejeon 34113, Korea; 3 Air Force Research Laboratory, 711th Human Performance Wing, Wright-Patterson Air Force Base, OH 45433, USA; steve.kim.13@us.af.mil; 4 Integrative Health & Performance Sciences Division, UES, Inc., Dayton, OH 45432, USA

**Keywords:** microfluidic thermal flowmeters, infusion pump calibration, microelectromechanical systems (MEMS), low flow rate measurement, human health, real-time sensors

## Abstract

This paper presents a microfluidic thermal flowmeter for monitoring injection pumps, which is essential to ensure proper patient treatment and reduce medication errors that can lead to severe injury or death. The standard gravimetric method for flow-rate monitoring requires a great deal of preparation and laboratory equipment and is impractical in clinics. Therefore, an alternative to the standard method suitable for remote, small-scale, and frequent infusion-pump monitoring is in great demand. Here, we propose a miniaturized thermal flowmeter consisting of a silicon substrate, a platinum heater layer on a silicon dioxide thin-membrane, and a polymer microchannel to provide accurate flow-rate measurement. The present thermal flowmeter is fabricated by the micromachining and micromolding process and exhibits sensitivity, linearity, and uncertainty of 0.722 mW/(g/h), 98.7%, and (2.36 ± 0.80)%, respectively, in the flow-rate range of 0.5–2.5 g/h when the flowmeter is operated in the constant temperature mode with the channel width of 0.5 mm. The measurement range of flow rate can be easily adjusted by changing the cross-sectional microchannel dimension. The present miniaturized thermal flowmeter shows a high potential for infusion-pump calibration in clinical settings.

## 1. Introduction

An infusion pump is one of the most commonly used medical equipment in clinics. It delivers medical fluids (e.g., nutrients, medications, and contrast agents [1]) into a patient’s circulatory system in controlled amounts and at precisely programmed rates. Due to its nature in delivering life-critical fluids, including high-risk medications, pump failures can lead to overdose, missed treatments or delayed therapy, thus significantly affecting patients’ health and safety. The U.S. FDA (Food and Drug Administration) reported approximately 56,000 cases of adverse events associated with infusion pumps from 2005 to 2009, including numerous injuries and deaths [2]. One of the best-known failures of the infusion pump originates from its inaccuracy when the pump is used without calibration. While the medical guidance for the infusion pump suggests accuracy with a 5% maximum uncertainty [3,4], modern precision personal medicine and healthcare require highly controlled medication with timely infusion [5]. For example, drug infusions for neonates, infants, and small children should be able to deliver multiple doses with high concentrations at low flow rates [6]. This medical demand is a significant challenge in the lower range (<3.0 g/h) since delivering the low-volume liquid requires more precise fluidic control than delivering the high volume [6,7,8]. Therefore, a flow-rate sensor capable of calibrating the infusion pump within the low range is imperative to ensure the safety of patients.

The standard calibration procedure for the infusion pump is based on the gravimetric method (GM) [1,9]. The GM uses a balance to measure the mass of injected fluid and calculates an SI-traceable flow rate (in the unit of g/h) through the mass change divided by injection time. Accurate GM requires a delicately controlled setup, such as draft shields, micro tubings, and dispensing needles, to counter any miscalibration from the fluid drift, evaporation, and bubbles [9]. Due to these requirements, the GM is typically performed in a well-controlled laboratory and is impractical to be deployed in hospitals, homecare, remote medical facilities, and en route care. An alternative to the GM suitable for these remote, small-scale, and frequent calibrations to the infusion pump is a great demand. Microelectromechanical system (MEMS) technology has shown to be significant for the realization of a miniaturized flowmeter platform [10]. The MEMS flowmeter explored to date includes optical [11,12], mechanical [13,14,15], and thermal flowmeters [16,17,18,19]. Amongst these, the thermal flowmeters consisting of a few elements (e.g., metallic heaters and temperature sensors) are the most common due to their simplicity [19,20] and high sensitivity [21] compared to non-thermal flowmeters. 

**Figure 1:**
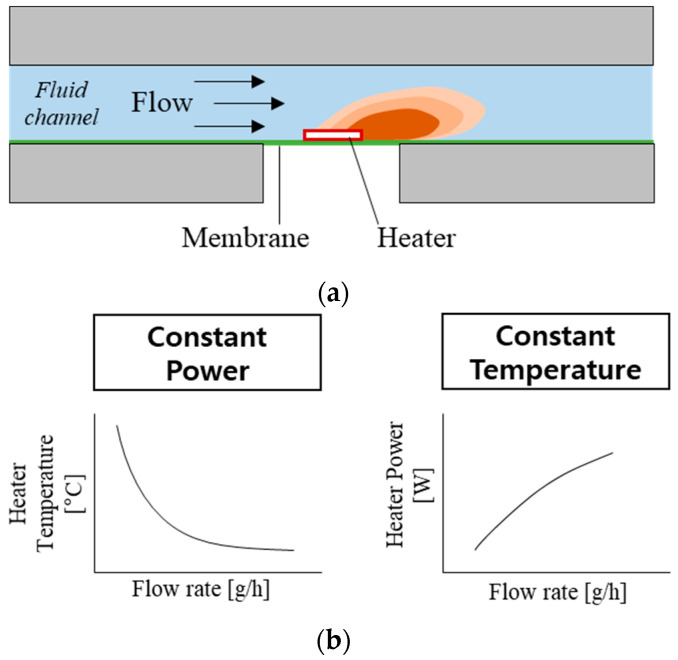
Working principle of the thermal flowmeter: (**a**) illustration showing the concept of flow-rate sensing; (**b**) two operation modes and their flow-rate sensing profiles.

The working principle of the thermal flowmeters is based on measuring the heat transfer from a heater to moving fluid (Figure 1) [17,18,22]. A simple thermal flowmeter can operate with a single heater by measuring (1) the heater temperature at constant heating power or (2) the power required to maintain the heater temperature constant (Figure 1b). However, one of the main challenges with the thermal flowmeters is slow response time (>1 min). Few flowmeters [18,22] have implemented heating elements on a wire or thin membrane to minimize the heat capacity and reduce their response time.

Typically, flowmeters have been applied to measure gas flow (in the typical range of 0.1~20 L/h) [23,24,25]. However, when they are used to measure liquids, the flow rate ranges in the high-flow regime (>50 g/h) [16,22,26]. A recent flowmeter [19] by D. Lee et al. demonstrated non-invasive flow measurement in a silicone tube, in which 5% uncertainty was achieved in the 0.1–100 g/h flow rate range. However, in their study, the flowmeter temperature sensors were positioned at the outer layer of the channel, resulting in a slow response time (~70 s), high power consumption (50 mW), and unintended heating of the fluid (~30 °C higher than the incident flow). 

Here, we present a MEMS flowmeter having a microfluidic channel and a thin membrane onto which a heater element is positioned to enable a rapid response time, low uncertainty, and low operating power. The GM was used as a reference to evaluate the device’s performance. The flowmeter dimension is 11.5 mm × 17.5 mm × 3 mm, suitable for many field-deployable liquid-flow calibration purposes.

## 2. Materials and Methods

### 2.1. Working Principle and Design

The present flowmeter uses two different sensing modes: Constant Power (CP) and Constant Temperature (CT) [22,24]. The CP mode (left side of Figure 1b) measures heater temperature depending on the flow rate, while the heater power remains constant. As the flow rate increases, heater temperature decreases due to the increased heat transfer. A one-dimensional model of the heater temperature is described as follows [27]:

(1) $$T_{h}=\frac{P}{k_{F}w_{h}\left(\frac{l_{h}}{\delta }+\sqrt{4k+\frac{v\delta^{2}}{4a^{2}}}\right)}$$

where *T_h_* is the heater temperature, *P* is the heat power, *k_F_* is the thermal conductivity of the fluid, *w_h_* is the heater width, *l_h_* is the heater length, *δ* is the boundary-layer thickness, *v* is the average flow velocity, *a* is the thermal diffusivity of fluid, *k =* ½ *+ k_Si_t_d_/k_F_δ* is the dimensionless factor, where *k_Si_* and *t_d_* are the thermal conductivity of the silicon substrate and the membrane thickness, respectively. Although the resistance of the heater varies slightly depending on the flow rate, it can be assumed that the total power remains constant. Then, the right side of the Equation (1) is constant except *v*: *T_h_* is inversely proportional to $\sqrt{v}$. 

In CT mode (right-side of Figure 1b), the external electric circuit adjusts the current so that the resistance (temperature) of the heating element is constant. As the flow rate increases, heating power needs to increase to compensate for heat loss and maintain the temperature. A one-dimensional model of heating power can be described from Equation (1) where *T_h_* is constant, and *P* is proportional to $\sqrt{v}$. The thin membrane underneath the heater lowers the *k* factor, resulting in a lower *P* and *T_h_* requirement while maintaining sensitivity. In addition, the small heat capacity is advantageous to reduce the time to reach a steady-state, resulting in a fast response time. 

**Figure 2:**
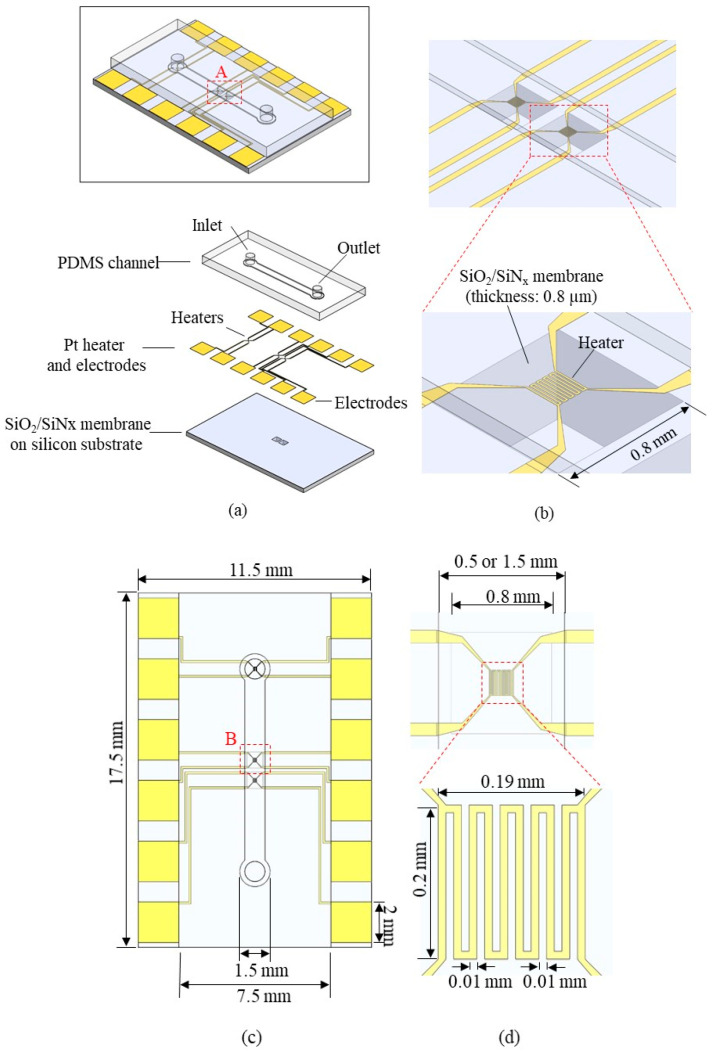
Schematic view of the flowmeter: (**a**) perspective view; (**b**) enlarged view of A in Figure 2a; (**c**) top view; (**d**) enlarged view of B in Figure 2d.

Figure 2 shows the schematic structure of the flowmeter. The flowmeter consists of a silicon substrate with a silicon dioxide/silicon nitride (SiO_2_/SiN_x_) membrane, a platinum (Pt) layer with heaters and electrodes, and a Polydimethylsiloxane (PDMS) channel layer (Figure 2a). Each heater is located on the silicon SiO_2_/SiN_x_ membrane of 800 μm × 800 μm × 0.8 μm (Figure 2b). The membrane is designed with high sensitivity for flow measurement by allowing heat to be transferred only to the fluid and not to the substrate. A thinner and broader membrane is more beneficial for a rapid response time and high sensitivity. Therefore, we selected membrane thickness as 0.8 µm, more than 500 times thinner than the silicon substrate, to minimize heat convection through the substrate and maximize the heat convention through the fluid. In addition, because of ultralow heat capacity, response time could be reduced significantly with this feature. To determine the appropriate membrane width, we fabricated 600 um-, 800 um-, and 1000 um-wide membranes (data not shown). We found that a 1000 um-wide membrane exhibited low fabrication yield and unstable mechanical durability during the flow measurement; thus, the 800 um-wide membrane was chosen as the optimal flowmeter platform. The composition of the membrane material consisted of 150 nm-thick SiN_x_ sandwiched by 300 nm and 350 nm-thick SiO_2_ layers, which configuration can balance the residual stress (compressive stress for the SiO_2_ and tensile stress for SiN_x_) for the robust mechanical characteristics and flat morphology of the membrane [28]. Heaters on both sides serve as reference sensors to monitor fluid temperature upstream and downstream (Figure 2c). The heater in the middle serves as a flow-sensing element to operate constant power mode or constant temperature mode. The heater has a total size of 0.19 mm × 0.20 mm and consists of serpentine electric lines with a width of 10 μm (Figure 2d). The heater is connected to 4 electrical connection lines for 4-wire remote sensing, able to serve as both heater and temperature sensor. Pt is chosen as the heater material because of good TCR (Temperature Coefficient of Resistance, 0.003729 K^−1^) [29] and biocompatibility suitable for biomedical flow sensing [27]. The PDMS channel height is 100 μm, and the width is 0.5 mm or 1.5 mm (Figure 2e). The narrow channel was selected as 0.5 mm, wide enough to fully cover the heater layer with 190 um-width. Then, the wider channel was chosen as 1.5 mm, three times wider than the narrow channel, to observe the effect of channel width. Finally, the channel height was selected as 0.1 mm due to fabrication ease using SU-8 2050. A 0.1 mm channel height exhibited a flow velocity of at least 10 mm/h at a 0.5 g/h flow rate, a crucial design factor because too-low flow velocity reduces flow-rate measurement sensitivity. The total length of the microchannel is 10 mm, and inlet/outlet holes are on both ends. The overall size of the substrate is 11.5 mm-width × 17.5 mm-height × 1.5 mm-thickness.

### 2.2. Fabrication Process

**Figure 3:**
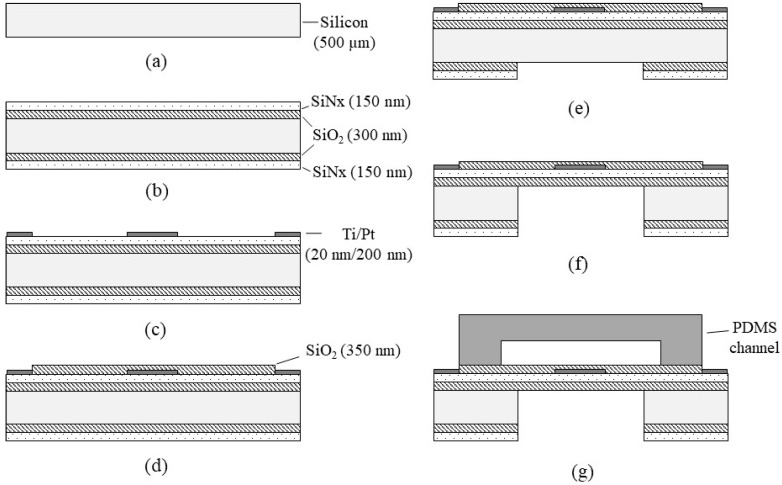
Fabrication process of the flowmeter: (**a**) preparation of a silicon substrate; (**b**) LPCVD SiO_2_/SiN_x_ deposition; (**c**) Ti/Pt electrode deposition and lift-off; (**d**) SiO_2_ deposition and etching; (**e**) backside SiO_2_/SiN_x_ etching; (**f**) silicon deep etching and dicing; (**g**) PDMS channel bonding.

The flowmeter was fabricated by conventional photolithography and bulk micromachining, as shown in Figure 3. Fabrication started from a 500 μm-thick silicon wafer (Namkang HI-TECH, Co. LTD, South Korea) with a diameter of 6 inches (Figure 3a). The 6-inch silicon wafer was double side polished, (100)-oriented, P-type, and 500 um thick. First, SiO_2_/SiN_x_ layers (300 nm of SiO_2_, and 150 nm of SiN_x_) were deposited onto the silicon wafer by LPCVD (Low-Pressure Chemical Vapor Deposition) (SiO_2_ layer: ~600 °C, SiN_x_ layer: >700 °C) (Figure 3b), respectively. Then, Ti(titanium)/Pt layers with a thickness of 20 nm/200 nm were deposited (by thermal evaporation) and patterned with conventional photolithography and the lift-off technique (Figure 3c). Another SiO_2_ layer (350 nm-thick) was deposited by PECVD (Plasma-Enhanced Chemical Vapor Deposition) and patterned (Figure 3d) on the electrode layer for electrical insulation of the heaters. The SiO_2_ layer was deposited at a low temperature (150 °C) during the PECVD process to eliminate detrimental effects on the metal film at high temperatures (750 °C) during the LPCVD process. Then, the backside SiO_2_/SiN_x_ was patterned (Figure 3e) to form a window for the deep etching process of the silicon substrate. Silicon deep etching was followed using DRIE (Deep Reactive-Ion Etching) (Figure 3f), resulting in thin membrane formation. The final wafer was then diced into unit devices by using a dicing saw. 

**Figure 4:**
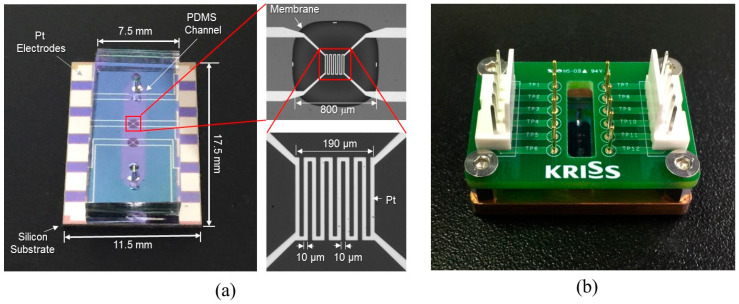
The fabricated flowmeter: (**a**) photograph of the fabricated thermal flowmeter with enlarged views of the membrane and the heater; (**b**) the flowmeter equipped with PCB jig for electrical connections.

The channel was fabricated by a PDMS micro-molding technique using a SU-8 negative photoresist. Briefly, SU-8 with a thickness of 100 μm was patterned onto the 4-inch diameter silicon wafer via conventional photolithography and used as a mold. Next, PDMS was prepared by mixing a base and curing agent with a mass ratio of 10:1. After degassing the mixture using a vacuum chamber for 10 min, PDMS was poured onto the mold and cured on the hot plate (100 °C for 15 min). The PDMS microchannel was then bonded onto the unit device after oxygen plasma treatment (Figure 3g). Figure 4a shows the fabricated flowmeter with enlarged views of the heater. The measured size of the fabricated flowmeter was 11.5 mm (width) × 17.5 mm (length) × 3 mm (height). A printed circuit board (PCB) holder having an array of probe pins was used to facilitate and secure the electric connection, as shown in Figure 4b.

### 2.3. Thermal Flowmeter Characterization Setup 

**Figure 5:**
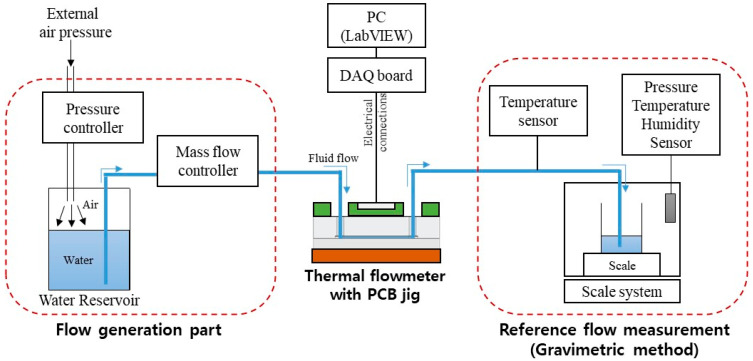
Experimental setup for the flowmeter characterization.

Figure 5 shows the experimental setup for evaluating the performance of the flowmeter. External air pressure to the water reservoir generated the water flow. A mass flow controller (MFC) (Bronkhorst mini Cori-flow M120) controlled the flow rate. The water flowed from the MFC through the flowmeter to the scale system with balance (Mettler-Toledo, XPE 206 DR). The evaporation trap was installed on the collecting glass to avoid liquid evaporation during the measurement. All system parts were connected using fluorinated ethylene propylene (FEP) tubing (PTFE 1/16″ OD × 1/32″ ID, Cole-Parmer, Vernon Hills, IL, USA). The tubing was connected to the needle at the scale. The tip of the needle was immersed in the water in the collecting glass vessel to avoid droplet formation and allow a continuous reading of the flow rate over time.

A Source Measurement Unit (NI-PXIe-4141, National Instruments, Austin, TX, USA) was electrically connected to four heater electrodes on the flowmeter for the flow measurement characterization. A customized program based on LabVIEW (National Instruments, Austin, TX, USA) controlled the applied current and monitored the electric signal from the flowmeter. The electric current was set at 3 mA for CP mode, and the voltage across the heater was measured to obtain heater resistance. Although the high current was advantageous for obtaining high sensitivity, there was an increased risk of bubble generation due to overheating, especially at a low flow rate. In CT mode, however, a closed-loop feedback algorithm adjusted the current to maintain the resistance 3 Ω higher than the resistance at room temperature, and input power was measured.

## 3. Results

**Figure 6:**
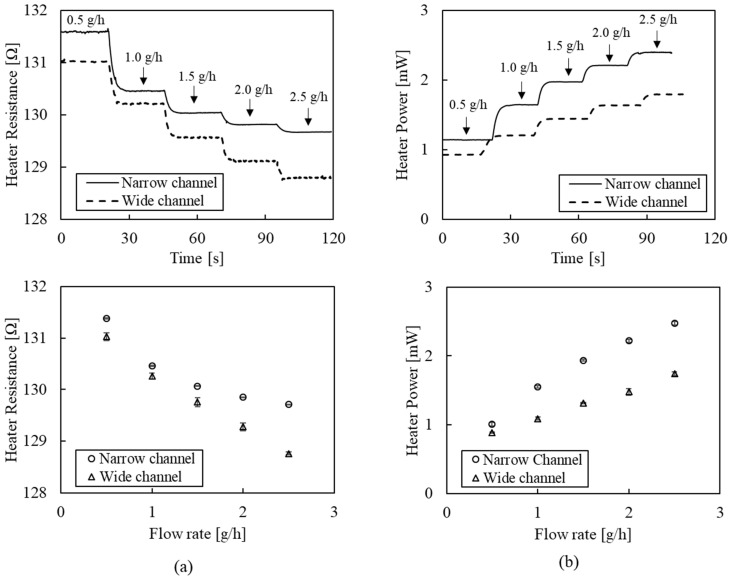
Flow-rate detection results of the flowmeter: (**a**) the CP mode; (**b**) the CT mode.

Figure 6 shows flow-rate detection results of the flowmeter using the CP or CT mode with a narrow (0.5 mm) or a wide (1.5 mm) channel. The CP mode (Figure 6a) detects a decrease in heater temperature as the flow rate increases. The top graph indicates distinguishable decrement changes in heater resistance as the flow rate increases from 0.5 g/h to 2.5 g/h with a step increment of 0.5 g/h. The response times, defined as the time interval between 10% and 90% of the entire span, were measured at 3.7 s ± 0.8 s and 3.4 s ± 0.3 s for the narrow and wide channels, respectively. We anticipate that these results are mainly associated with the mass flow controller’s response time. The bottom graph shows the heater resistance depending on the flow rate, where the average and standard deviation were obtained from five replicated tests. The wide channel showed better linearity (99.5%) and sensitivity (−1.104 Ω/(g/h)) than the narrow channel (linearity: 93.1%, sensitivity: −0.790 Ω/(g/h)). While having a higher sensitivity in the low range (under 1.0 g/h) than the wide channel, the narrow channel exhibited a nonlinear behavior at flow rates over 1.5 g/h. Equation (1) explains that the heat-transfer rate is related to the flow velocity rather than the flow rate. Therefore, the narrow channel, having a higher velocity than the wide channel at the same flow rate, will saturate earlier. In the case of the wide channel, the sensor signal does not tend to saturate in the 0.5–2.5 g/h range. However, a wider channel width than heater size would cause a decreased sensitivity. Therefore, optimal design of geometric dimensions, including the channel width and heater size depending on the target flow range, is crucial to acquiring a high sensitivity signal. 

The CT mode (Figure 6b) measures an increased heater power while maintaining the constant temperature under fluid flow. The top graph indicates distinguishable changes in heater power as the flow rate increases from 0.5 g/h to 2.5 g/h with a step increment of 0.5 g/h. The response times were measured at 3.4 s ± 0.3 s and 4.1 s ± 0.7 s, for the narrow and wide channels, respectively. The bottom graph shows heater power depending on the flow rate, where the average and standard deviation were obtained from five replicated tests. The narrow and wide channels showed more than 98% linearity (98.7% and 99.8%). The sensitivity of the narrow channel (0.722 mW/(g/h)) was 1.8 times higher than that of the wide channel (0.421 mW/(g/h)) due to its higher flow velocity. While the CT mode shows high linearity, it requires a precise feedback system that maintains heater temperature, involving more equipment and costs.

**Figure 7:**
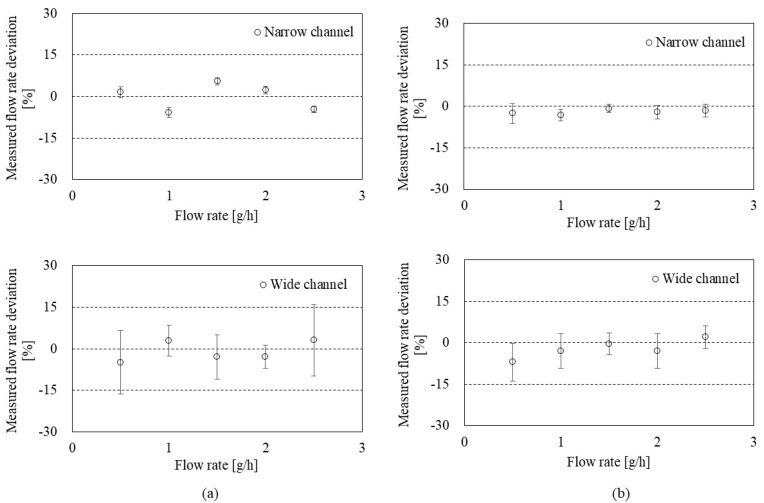
Uncertainty evaluation of the flowmeter in: (**a**) the CP mode with the narrow channel (**top**) and the wide channel (**bottom**); (**b**) the CT mode with the narrow channel (**top**) and the wide channel (**bottom**).

To evaluate the accuracy of the flowmeter, Figure 7 shows the deviation and uncertainty depending on the flow rate in the CP mode (**a**) and the CT mode (**b**). S.H. Lee et al. [30] provided a mathematical model to calculate the deviation and uncertainty. Briefly, the deviation is the relative difference (%) calculated by the measured flow rate (from the present flowmeter) and the reference flow rate (from the GM). Then, the uncertainty of the flowmeter was calculated by Type A uncertainty (from the repeated observation) and Type B uncertainty (from the calibration reports for weighing system, timer, etc.). As a result, the CP mode (Figure 7a) showed relative deviations of less than 10% in both narrow and wide channels. Average uncertainties in 0.5–2.5 g/h ranges were 1.52 ± 0.40% and 8.44 ± 3.75% in the narrow and wide channels, respectively. 

The CT mode (Figure 7b) also showed less than 10% relative deviations in both narrow and wide channels. Average uncertainties in 0.5–2.5 g/h ranges were 2.36 ± 0.80% and 4.90 ± 2.44% in the narrow and wide channels, respectively. The uncertainty of the flow rate measurement in CT mode can vary depending on the performance of the feedback system (e.g., frequency and resolution).

The narrow channel showed the optimal performance for both CP and CT modes for measuring flow rate in the range of 0.5–2.5 g/h, where the measurement uncertainty was less than 3%. This accuracy performance is more than five times better than that of the commercial portable infusion pumps having uncertainties of approximately 15% [31]. Since the output signal is directly relevant to the flow velocity, it is possible to tune the appropriate flow range depending on the target application by modifying the microchannel sizes (width and height). For example, if the channel height exceeds 100 μm, the flowmeter will detect a flow rate sensitively more than the 3.0 g/h flow range. On the other hand, a channel height lower than 100 μm would expect to provide a sensitive signal at a low flow range under 0.5 g/h.

## 4. Discussion

It is essential to consider several factors affecting the flowmeter characteristics to improve measurement accuracy. For example, temperature stability is a crucial factor affecting measurement uncertainty. When the ambient temperature changes, the heat transfer to the fluid changes, increasing the flow-measurement uncertainty. A change in the water temperature causes changes in the amount of heat transferred from the heater to the water. For example, a 1 °C change in ambient temperature can result in approximately 0.3 g/h offset, significantly complicating the flowmeter accuracy. Therefore, minimizing the temperature change around the injection fluid and the flowmeter is necessary. One solution to the temperature variation issue is using a thermostat container that provides constant environmental temperature within 0.1 °C stability to improve measurement repeatability, deviation, and uncertainty. 

Noise level is an essential factor for sensor performance, especially for a signal-to-noise ratio and detection limit rather than accuracy. The typical way to minimize the effect of noise on the flowmeter accuracy is to average measured signals and baselines for a longer time so that the measurement data are close to the true value.

High power (or high temperature) at the heater is beneficial for increasing the sensitivity of the sensor, but it can heat the substrate, making the hysteresis response of the sensor worse. It can also damage biological samples injected by the pumps. Therefore, selecting an appropriate heating power is required. In this study, 3 mW of heater power was used considering the sensitivity and hysteresis of the sensor. 

One of the critical issues with sensor research is the baseline drift. Even commercial off-the-shelf sensors suffer from drift, and frequent calibration is required to correct this. Several sensor works have adopted correction equations to compensate drift phenomenon for solving this issue. However, for thermal-type sensors such as the present flowmeter, the drift can occur when ambient temperature variation is significant as a signal from the heater only depends on the temperature. Therefore, we anticipate that signal drift may be minimal as long as the temperature is constant.

The flowmeter could detect air bubbles. The air bubbles inside a fluid channel change the sensor signal dramatically due to a thermal property discrepancy between water and air. The size of the bubble can also be roughly detected through the duration of the signal. Since excessive bubble injection could have fatal consequences for the patient, bubble detection is one of the important functions required to monitor drug injection status.

The present flowmeter aimed to monitor drug infusion for medical use, thus focusing on flow-rate measurement of water that is the solution for most infusion fluids (e.g., intravenous fluids). However, this flowmeter can expand its application to other fields (e.g., micromixers and chemical reactors) by characterizing the corresponding target chemicals such as isopropyl alcohol and ethanol. In addition, the present flowmeter can serve as a gas-velocity sensor by characterizing the heater responses to air flows. Since the thermal properties of each material are different, the flow-rate correction is required.

## 5. Conclusions

A MEMS flowmeter with a microfluidic channel is designed and fabricated via micromachining to measure low flow rates for infusion-pump calibration. The MEMS flowmeter consisted of a silicon substrate, a Pt heater layer on a SiO_2_/SiN_x_ thin-membrane, and a PDMS mold, with a size of 11.5 mm (width) × 17.5 mm (length) × 3 mm (height). The standard GM is applied to evaluate the performance of the flowmeter in two different modes (the CP and CT modes) and two different channel widths (0.5 mm and 1.5 mm). The flowmeter in the CT mode with a 0.5 mm-wide channel shows the optimal performance where the uncertainty is five times better than the commercial portable infusion pump in the 0.5–2.5 g/h range. In addition, the present flowmeter shows significant improvement in response time, power consumption, and flow-rate reading at low flow volume compared to previous flowmeters. The present flowmeter provides the potential to use as an on-chip on-demand or continuous self-calibration tool for the safe and effective use of infusion pumps in clinical applications.

## Data Availability

All data generated or that appeared in this study are available upon request by contact with the corresponding author. Furthermore, the models and code used during the study cannot be shared as the data also form part of an ongoing study.
